# Green tea and metabolic syndrome: A 10-year research update review

**DOI:** 10.22038/IJBMS.2021.52980.11943

**Published:** 2021-09

**Authors:** Elahe Esmaeelpanah, Bibi Marjan Razavi, Hossein Hosseinzadeh

**Affiliations:** 1 Department of Pharmacodynamics and Toxicology, School of Pharmacy, Mashhad University of Medical Sciences, Mashhad, Iran; 2 Targeted Drug Delivery Research Center, Pharmaceutical Technology Institute, Mashhad University of Medical Sciences, Mashhad, Iran; 3 Pharmaceutical Research Center, Pharmaceutical Technology Institute, Mashhad University of Medical Sciences, Mashhad, Iran

**Keywords:** Diabetes, Dyslipidemia, Green tea, Hypertension, Metabolic syndrome, Obesity

## Abstract

Metabolic syndrome (MetS) has turned into a prevalent condition that has imposed a tremendous financial strain on public health care systems. It is believed that the MetS consists of four main factors (hypertension, dyslipidemia, hyperglycemia, and obesity) and may lead to cardiovascular events. *Camellia sinesis*, in the form of green tea (GT), is one of the most consuming beverages worldwide. Catechins are the dominant component of green tea leaves. Epigallocatechin gallate has the maximum potency. GT has been widely used as a supplement in various health conditions. As the oxidative stress pathway is one of the probable mechanisms of MetS etiologies and GT beneficial effects, GT may be a novel strategy to overcome the MetS. This review aims to reveal the probable pharmacological effects of GT on MetS. The last 10-year original articles on MetS parameters and GT have been gathered in this review. This manuscript has summarized the probable effects of green tea and its catechins on MetS and focused on each different aspect of MetS separately, which can be used as a basis for further investigations for introducing effective compounds as a way to interfere with MetS.

It seems that GT can reduce MetS parameters commonly via anti-inflammatory and anti-oxidative mechanisms. Further clinical trials are needed to confirm the use of GT and its constituents for the treatment of MetS.

## Introduction

Metabolic syndrome (MetS), which has also been named as “syndrome X”, “Reaven syndrome” and “insulin resistance syndrome”, was described by Reaven in 1988 for the very first time (1, 2). It is estimated that about 10% to 40% of the world population is suffering from the MetS. Excessive nutrient intake, sedentary lifestyle, and genetic factors are amongst predisposing causes to afflict people with MetS (3). 

There is no complete agreement on the exact definition of MetS. According to the latest harmonized definition, MetS is a set of clinical signs including hypertension, dyslipidemia (comprises elevated triglycerides or reduced high-density lipoprotein (HDL) level), hyperglycemia, and central obesity. MetS diagnostic criteria is based on having 3 or more of the above components (1, 3). The implication to MetS would accelerate atherosclerosis which amplifies the risk of cardiovascular events. The etiology of MetS is not fully understood, but it is thought to be a proinflammatory and prothrombotic state (1). Treatment is based on prevention and lifestyle modification (3). In some of the previous studies, the effects of different herbals such as *Vitis vinifera *(4)*, Crataegus pinnatifida* (5), *Rosmarinus officinalis *(6), *Allium sativum *(7), *Nigella sativa *(8), *Citrus paradisi* (9), *Capsicum annuum *(10), *Aloe vera *(11), *Berberis vulgaris *(12), *Persea americana *(13), *Silybum marianum *(14), *Garcinia mangostana *(15), *Crocus sativus *(16), *Cinnamomum verum* (17) and rutin (18) on MetS parameters had been reviewed.

The fresh leaves of *Camellia sinensis* are being consumed as white, green, oolong, and black tea based on the oxidation degree (19). Green tea (GT) is a universal herbal tea mostly used in Asia, some parts of North Africa, the United States, and Europe. This type of tea is known to have the most significant impact on human health (20, 21).

**Figure 1:**
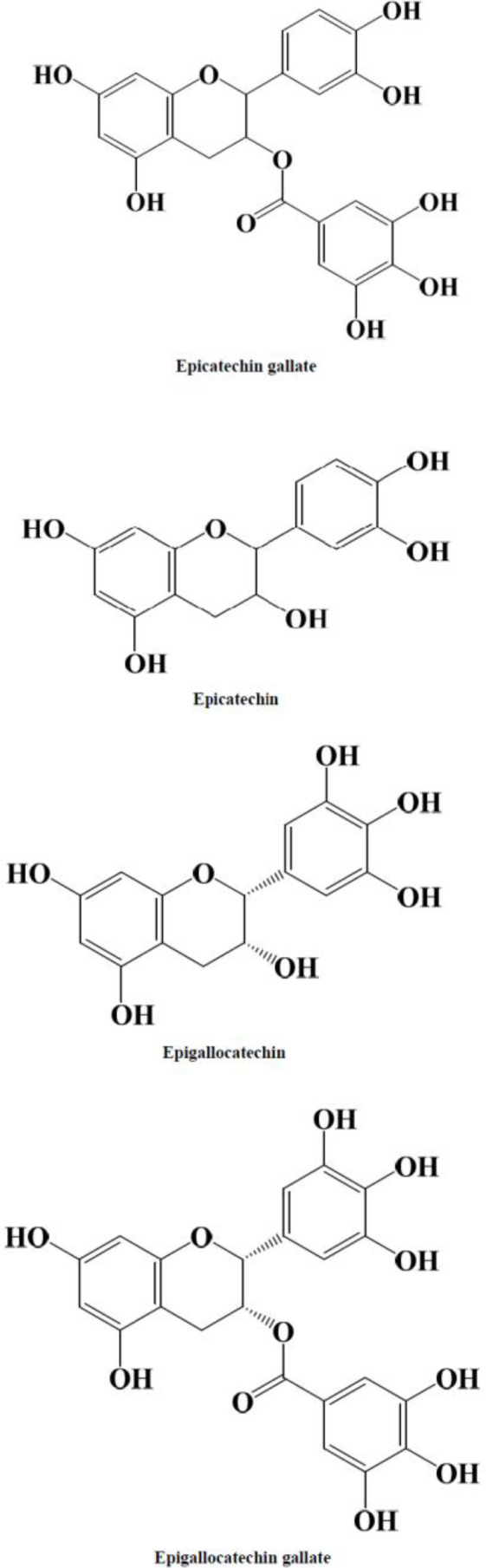
Chemical structures of green tea catechins

GT polyphenols or so-called catechins are known to be the main constituent in most of the beneficial effects of GT. Catechins include epigallocatechin gallate (EGCG), epigallocatechin (EGC), epicatechin gallate (ECG), and epicatechin (EC). The chemical structure of these compounds is shown in Figure 1. EGCG is the major component of GT polyphenols (21-23).

GT was included in “therapeutic compounds” of ancient times, and there are large sets of data on the medical properties of GT and its catechins. GT and its catechins have been studied in some diseases include obesity (24), diabetes(25), cardiovascular disease (26), dyslipidemia(27), cancer (28, 29), neurodegenerative disorders (30-32), antimicrobial (33) and antitoxin effects (34). Most of these beneficial properties are known to mediate through anti-oxidant properties (35).

**Figure 2:**
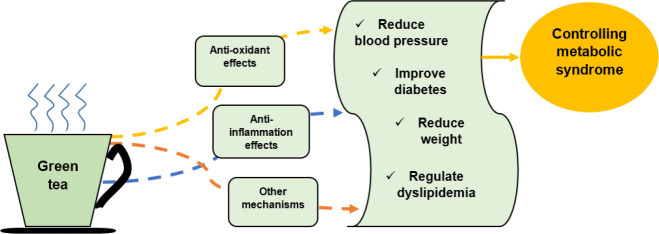
Schematic description of protective mechanisms of green tea on metabolic syndrome

Due to the increasing incidence of MetS in the last decades and because it is probably oxidative stress is one of the underlying mechanisms for expanding the disease (1, 36), it is thought that adding herbal compounds with anti-oxidant properties such as GT and it’s catechins to diet, may prevent or improve MetS. Moreover, the beneficial effects of GT on MetS factors have been widely investigated in the literature. Therefore, the data that have been reviewed here attempt to provide information about the effects of GT and its catechins on MetS components. The schematic description of the main GT’s effects is shown in Figure 2.

## Materials and Methods

The databases PubMed, Scopus, and Web of Science were used for hunting the related articles for the last decade (2009-2019). Due to the popularity of GT’s beneficial effects, there were extensive articles on this topic; therefore, the authors have decided to include the newest experiments from 2009 onwards in this manuscript. 

The keywords “Green tea”, “*Camellia sinensis*” and “*Thea sinensis*” were used for GT and the keywords epicatechin, epicatechin-3-gallate, “epicatechin gallate”, epigallocatechin, epigallocatechol, epigallocatechin-3-gallate, and “epigallo-catechin gallate” were used for the catechins.

The keywords for MetS include hypertension, “blood pressure”, hypotensive, anti-hypertensive, diabetes, hyperglycemia, insulin, hypoglycemic, anti-hyperglycemic, anti-diabetic, “blood glucose”, dyslipidemia, hyperlipidemia, “high cholesterol”, “high triglyceride”, hypercholesterolemia, hypertriglyceridemia, atherogenic, atherosclerosis, obesity, overweight, appetite, anti-obesity and “weight loss”.

After finding 500 articles, the studies which used the combination of GT and other compounds or used modified-GT or the catechins of other herbs were excluded in this review article. The remaining original articles in animal or clinical models were selected exclusively. 

***Effects on metabolic syndrome***

*Animal studies*

Animal models of MetS were induced through different mechanisms as drug-induced (37) and regimen-induced (38). Blood glucose (BG), triglycerides (TG) level, High density lipoprotein (HDL), low-density lipoprotein (LDL), blood pressure (BP), body weight (BW), and food intake were measured in the majority of studies as the most common indicators of MetS. 

All of the animal experiments were done on rats except for one that used male mice as the MetS model. In mice, treatment with EGCG as a part of a high fat/Western-style diet (0.32%) for 17 weeks significantly attenuated body weight gain, BG, insulin resistance, and plasma cholesterol level in C57BL/6J mice (38). 

Green tea extract (GTE) 200, 300 mg/kg, orally treated for 9 weeks decreased BP and improved the metabolic profile, adjusted adiponectin level, and adiponectin receptor gene expression and increased PPARα and PPARγ gene expression in MetS rat model (39-41). In another study, GT powder (10%) and GT ethanoic extract (5%) showed beneficial effects against hypercholesterolemia and hyperglycemia after 8 weeks (42). Razavi *et al.* found that GT aqueous extract at doses 25, 50, and 100 mg/kg/day (IP) for 11 days could modulate olanzapine-induced MetS parameters in rats (37).

*Clinical studies*

FBS, BP, lipid profile, and BW were amongst factors that were measured as MetS indicators (43-46). Water was used for the control group in some of the studies (43-45, 47). 

In a series of randomized controlled trials with GT beverage (4 cups per day) and GTE (2 capsules per day) for 8 weeks in 35 obese subjects with MetS, plasma anti-oxidant capacity and whole blood glutathione were significantly increased. Likewise, plasma serum amyloid alpha (which is an independent risk factor for cardiovascular disease), BW, and BMI were significantly reduced, and lipid peroxidation was lowered (43, 44, 47). 

An epidemiological study based on a self-administered questionnaire in the Korean population (n=15568, aged 19-65 years) revealed that regular consumption of GT beverage was inversely associated with MetS (48).

In a randomized control trial of 70 women with a confirmed diagnosis of MetS, anthropometric indices, BP, BG, and lipid profile significantly improved after drinking 200 ml of GT 3-times per day for eight weeks (45).

In an interventional study, Senger *et al.* concluded that consumption of three cups of GT (as 1 g sachets) for 60 days, induced weight loss, reduced BMI and waist circumference in 45 elderly with MetS, although not changing their lipidic and glycemic profile (46).

***Effects on dyslipidemia***

*Animal studies*

Different models have been developed to assess the beneficial effects of GT. Serving an atherogenic diet that contains high fat, cholesterol, and sugar was used in a series of work (27, 49-51). Intraperitoneal (IP) injection of EGCG (100 mg/kg/day) from day 31 of a 45-day atherogenic regimen in rats significantly alleviated inflammation markers such as C-reactive protein (CRP), which was increased due to the atherogenic diet (51). In a similar study with rats feeding an atherogenic diet followed by 7-15 days of GT catechin (100 mg/kg IP), the hepatic anti-oxidant profile improved (50). 

Adding dextran sodium sulfate (DSS) to the drinking water of rats feeding an atherogenic diet, leading to dyslipidemia, increasing inflammation, and hepatic toxicity markers, which was diminished by GTE supplementation (0.2% in the diet) for 4 weeks (27). 

Treatment of rats with sodium fluoride (25 mg/kg) for 4 weeks is another model which been used in the study of Miltonprabu and Thangapandiyan*.* Pre-administration of EGCG (40 mg/kg) protected the intoxicated rats against dyslipidemia and cardiotoxicity (52).

Apo lipoprotein-E knockout mice, which is a well-established model for human atherosclerosis, have been used along with a high-fat diet (HFD) (53-55). The intragastric administration of EGCG (40 mg/kg/d for 18 weeks) significantly suppressed atherosclerotic plaque formation and lipid accumulation in the liver and also modulated dyslipidemia (53). In a similar study, GT polyphenols (3.2 or 6.4 g/l in drinking water for 15 weeks) significantly suppressed the atherogenesis rate through lipid metabolism improvement and increasing the expression of hepatic PPARα and autophagy markers in the mice vessel walls (55). Cai *et al.* used *Porphyromonas gingivalis* as an auxiliary factor for accelerating atheroma formation in the proximal aorta. EGCG supplementation (via drinking water for 15 weeks) suppressed atherosclerosis lesions through anti-inflammatory and anti-oxidative effects (54).

Furthermore, EC (0.1% w/w in diet) alleviated atherosclerosis development and restrained progression from mild to severe lesions with no effect on dyslipidemia in apoE*3-Leiden mice fed an atherogenic diet probably through anti-inflammatory pathways (49).

LDL receptor knockout murine model fed a hypercholesterolemic diet has been used by Minatti *et al.* GTE at low doses (50, 100 mg/kg/day by gavage) reversed endothelial dysfunction which has led to reduction of atherosclerosis progression along with lowering plasma TG and Monocyte chemoattractant protein-1 (MCP-1) level (56).

Other studies about the effects of GT on dyslipidemia parameters are shown in Table 1.

*Clinical studies*

TG, HDL, LDL, and total cholesterol levels were the most prevalent indicators of dyslipidemia which were measured in the available clinical studies about GT.

The first study was a randomized, multicenter placebo-controlled, double-blind study of 30 patients with hypertriglyceridemia, aged 18-55 years old, who were given a total daily dose of 100 mg EC in the form of 25 mg capsules for 4 weeks. It is concluded that EC could have an integrated positive metabolic response which may be beneficial in cardiovascular disease risk (57).

Another study was done with 250 mg capsules of GTE and placebo for 8 weeks in 33 patients, aged 21-71 years old, with a low-fat diet (25-35% of total calories and 200 mg of cholesterol per day) as a prospective, double-blind crossover study. LDL-cholesterol levels and total cholesterol were significantly reduced, which demonstrates the profitable effects of GT (26).

One double-blind, placebo-controlled trial in 56 obese, hypertensive subjects caused a remarkable attenuation in LDL, total cholesterol, and TG and increased HDL after giving 1 GTE capsule daily for 3 months (25). EC (1 mg/kg 30 min before tests) lowered TG concentrations by boosting lipid oxidation in 20 normal weight and overweight subjects in a pilot crossover, open-labeled study (58). GTE reduced LDL but showed no effect on total cholesterol, TG, and HDL after a 6-week GTE consumption in obese and overweight women (59). In 120 overweight women, daily GT capsules for 12 weeks remarkably lowered total cholesterol and LDL levels in a double-blind study (60). In another clinical trial, consuming the GT formula for 12 weeks in overweight or obese subjects caused significant effects on total cholesterol and LDL levels [79]. Moreover, a remarkable improvement in TG and HDL levels was reported after GTE consumption (500 mg TDS) for 16 weeks in 92 type-2 diabetes (T2D) and dyslipidemia subjects in a double-blinded, randomized and placebo-controlled clinical trial (61)

***Effects on Obesity***

*Animal studies*

Obesity-related effects that are commonly reported in the studies include reduction of BW (38, 62-73), decline in fat mass (67, 71, 72, 74, 75) or body organs weight (68, 69), and modifying gut microbiomes (67, 76).

**Figure 3:**
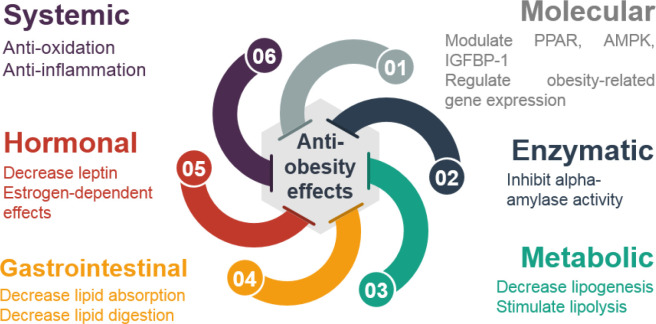
Different mechanisms for anti-obesity effects of green tea

The mentioned effects are thought to occur via different mechanisms, as shown in Figure 3. Molecular mechanisms consist of modulating PPAR (68, 74, 77), 5’ AMP-activated protein kinase (AMPK) (70), or insulin-like growth factor-binding protein 1 (IGFBP-1) (78). The effects on obesity-related gene expression (62) are also considered to be another molecular mechanism. Moreover, the systemic effects of GT consist of anti-oxidant (69) and anti-inflammatory effects (38, 62, 64, 65, 67, 71, 79) and hormonal mechanisms through decreasing leptin level (72, 80, 81) and estrogen-dependent effects (62) have been considered in several articles. The enzymatic mechanisms such as inhibiting alpha-amylase activity (82), metabolic mechanisms such as reducing lipogenesis (67, 70) and stimulating lipolysis (64, 71, 74), and gastrointestinal mechanisms through decreasing lipid absorption (38, 72) and digestion (66) are amongst other probable effects of GT.

Most of the studies used mice fed with the HFD diet as an obesity model. Except for several studies that used rats (68, 70, 73, 83) and a work by Huang that used chickens as a model (74). 

The preventive role of GT in obesity was assessed in several studies. It is thought that supplementation with GT polyphenols for 3 weeks, may prevent obesity in mice fed with HFD due to the prebiotic-similar activities (76). IGFBP-1, a novel molecule in obesity prevention, was elevated in white adipose tissue by adding GT to drinking water for 14 weeks (78). Other study protocols have been reviewed in Table 2. 

*Clinical studies*

In a single-blind, placebo-controlled, parallel clinical trial on a population of the north of Iran, GT (a cup of GT half an hour after breakfast and lunch for 12 weeks) was introduced as an efficient option in obesity owing to the remarkable effects on BW, BMI, waist and hip circumferences (84). A 12 weeks’ treatment with high dose GTE (containing 857 mg EGCG) in women with central obesity, made a remarkable weight loss in a randomized, double-blind study (85). Another 12 weeks clinical trial by consuming the GT formula in overweight or obese subjects showed greater weight loss and body fat mass reduction than the control group (86). Using 500 mg GT tablets for 8 weeks in overweight men caused a significant attenuation in BW, BMI, and waist-hip ratio in a semi-experimental study (87)

Several studies were done to understand the catechins’ effects. In a pilot crossover, the open-labeled study of 20 normal weight and overweight subjects were given 1 mg/kg of EC 30 min before the tests, the fat oxidation increased following a meal which was more vigorous in overweight subjects (58). Moreover, a low dose of ECGC (300 mg orally for 3 days) intensified fat oxidation in obese men after a meal. However, it did not show any effects on energy consumption in a randomized, placebo-controlled, double-blind crossover trial (88). In another study, 14 healthy volunteers given 300 ml GT with breakfast reported remarkably higher satiety and lower desire to eat rather than the control group in a randomized controlled trial (89). An 8-week-period study with GT in 58 T2D patients with BMI ≥ 25 showed a significant reduction in weight and BMI in a double-blind randomized clinical trial (90). Another randomized clinical trial also reported considerable mitigation in BW, BMI, and waist circumference in T2D patients drinking 4 cups of GT daily (91).

One of the studies was done on a special population i.e., overweight polycystic ovary syndrome (PCOS) women. In this double-blind, placebo-controlled, randomized clinical trial on overweight PCOS women, GT tablets (twice daily) reduced BW significantly 12 weeks after the intervention (92).

On the other hand, some of the scientific papers revealed little to no effect of GT on obesity. For instance, EGCG (300 mg/d in the diet for 12 weeks) had no effects on energy-restricted diet-induced adiposity deprivation and weight-loss-induced alterations in cardiometabolic risk factors in obese Caucasian women in a randomized, double-blind, placebo-controlled study (93). A 6 weeks’ intervention with GTE effectively elevated leptin levels, although not affect other overweight-related biochemical markers in obese and overweight women (59). In another randomized trial, GT supplementation (3 cups/day for 10 weeks) did not demonstrate any effects on apelin and orexin-A in obese women (94). Finally, In a controlled, randomized clinical trial in overweight or obese subjects, GT (5 g/day for 8 weeks) had no remarkable effects on leptin and paraoxonase (PON-1), which is an indicator of inflammation (95).

***Effects on diabetes***

*Animal studies*

GT has valuable effects on BG and insulin levels and improves insulin resistance. The underlying mechanisms for these effects were discussed in several studies that are being introduced briefly. 

As a result of diabetes-induced regimens, the activation of insulin signaling cascade members (such as insulin receptor, insulin receptor substrate-1 (IRS1), Protein kinase B (Akt), and Extracellular Signal-Regulated Kinases 1 and 2 (ERK1/2) diminished and negative modulators (such as Protein Kinase C (PKC), IkappaB kinase beta (IKK), c-Jun N-terminal kinase (JNK), and protein tyrosine phosphatase 1B (PTP1B) augmented. Supplementation with EC could prevent these alterations (96). Moreover, the downregulation of the inhibitory molecules PKC, IKK, JNK, and PTP1B by EC led to the attenuation of obesity-related insulin resistance (97). Supplementation with EGCG (for 12 weeks) diminished BG and insulin levels through restoring Akt activity and Glucose transporter type 4 (GLUT4) expression and enhancing AMPKα activation in skeletal muscle (98). Other experiments which discussed the beneficial effects of GT on these molecules are shown in table 3 (99).

Some studies showed that inflammation is involved in insulin resistance (79, 100-104). In one study, the regulator of G-protein Signaling 10 (RGS10) deficiency, which could intensify HFD-induced insulin resistance and inflammation, was restrained by GTE (100). Otton *et al.* concluded that MicroRNA (miR)-335 links inflammation to impaired metabolism in adipose tissue, which was decreased by GT treatment (500 mg/kg for 12 weeks) in HFD-fed mice (102).

Oxidative stress is another possible mechanism for GT’s effects. In one study, dietary EGCG reduced advanced glycation end products (AGE) level, inhibited the AGE receptor, and enhanced reduced glutathione/oxidized glutathione ratio (GSH/GSSG ratio) (69). There are other studies in this regard that are mentioned in table 3 (103, 105-107). In another experiment, GT treatment restored oxidative and inflammation parameters in the retina, and it was suggested for the treatment of diabetic retinopathy (108).

One study introduced the immune system in the favorable effects of GT. EC regulated immune function (by increasing interleukin-10 level), reduced pancreatic insulitis, and also improved pancreatic islet mass, which led to type-1 diabetes prevention(109).

EGCG boosted insulin clearance by hepatic Insulin-degrading enzyme (IDE), which led to improving insulin resistance in a dose-dependent manner (110). Furthermore, EGCG (10 μM) given to diabetic pregnant mice resulted in a lower rate of neural tube defect incidence. Therefore, it is thought that GT can attenuate hyperglycemia-induced teratogenic effects (111). The studies about the correlation between GT and diabetes are shown in Table 3.

*Clinical studies*

The results of the clinical studies in this area are contradictory. Some studies reported valuable effects from GT on diabetes parameters.

In a double-blind, placebo-controlled trial in 56 obese, hypertensive subjects, daily supplementation with 1 capsule of GTE (379 mg) for 3 months led to a significant reduction in fasting serum glucose, insulin level, and insulin resistance. Also, mitigation in serum (Tumor Necrosis Factor-α) TNF- α and CRP level and an increase in total anti-oxidant status was observed (25). Another study revealed that GTE (500 mg TDS for 16 weeks) remarkably improved insulin resistance and augmented glucagon-like peptide 1 in 92 T2D and dyslipidemia subjects in a double-blinded, randomized, and placebo-controlled clinical trial (61). One cross-sectional observational study on 75 healthy volunteers and 75 T2D patients concluded that GT could lower BG in diabetic subjects (112).

In a comparative study between usual and high doses of GT in healthy adolescents, higher doses of GT reduced postprandial blood glucose (PBG) level. Hence, higher doses were recommended for better control of PBG (113). In another cross-sectional study in 35 male volunteers in Japan, the lowering effects of 3% GT on fasting blood glucose (FBG) and fructosamine tended to be higher than 1% concentration. However, the frequency of drinking did not affect these parameters (114). 

EGCG used as dietary supplementation in the third semester of 472 pregnant women with Gestational diabetes mellitus successfully improved maternal diabetic parameters and mitigated the number of neonatal incidents in a double-blind randomized controlled trial (115).

In a pilot crossover, open-labeled study, 1 mg/kg EC was given to 20 normal weight and overweight subjects 30 min before the tests. EC remarkably reduced postprandial plasma glucose (PPG), which was more prominent in overweight subjects (58).

In a double-blind, crossover design short term GTE consumption (1050 mg/day for 7 days) in 11 sedentary subjects, overweight men did not affect PBG at rest, but reduced PPG after exercise, which is thought to be through alterations of glucose uptake in skeletal muscle (116). One randomized, double-blind placebo-controlled trial of GTE (300 or 600 or 900 mg/day) in 51 T2D patients indicated that stimulation of Soluble receptor for advanced glycation end products (sRAGE) production, which led to the receptor for advanced glycation end-products (RAGE) ligand inhibition is one of the underlying mechanism for EGCG effects in diabetes (117). One more mechanistic study targeted increased adiponectin level as the main mechanism for the T2D controlling effect of an 8-week-period of GT consumption which led to improving glycosylated hemoglobin A1c (HbA1C) levels in 58 T2D patients with BMI≥25 in a double-blind randomized clinical trial (90). In a two-group, double-blind, placebo-controlled, randomized clinical trial GT tablets (twice daily) reduced fasting insulin in 60 overweight PCOS women after 12 weeks (92). Daily GT capsules for 12 weeks reduced fasting glucose in 120 overweight women in a double-blind study (60). 

On the other hand, some experiments showed no to less effect from GT consumption. In a randomized controlled trial on 40-65 years old overweight or obese male subjects, no observable effect on glucose tolerance, insulin sensitivity, or insulin secretion was reported from EGCG capsules (400 mg BD for 8 weeks)(118). In another randomized controlled trial, 14 healthy volunteers were given 300 ml of GT or water together with the same breakfast. Postprandial glucose levels 120 min after ingestion GT containing meal were higher. Moreover, there were not any significant differences in serum insulin levels and glucose/insulin area under the curve (AUC) (89).

In a survey of Shanghai health study, data on type and amount of tea drinking habits were collected and the incident of T2D was assessed through follow-up surveys. This survey revealed that the risk of T2D was higher in the current GT drinkers (119). In one randomized human cohort of GT (3 cups daily for 14 weeks followed by a 2-week washout period) in pre-diabetic subjects, it is concluded that the timescale was not enough to exhibit GT effects on fasting plasma glucose and HbA1c (120). 

***Effects on hypertension***

*Animal studies*

There are 2 similar studies by Gomez *et al.* about the effects of EC on hypertension. The first study revealed that 4 weeks oral administration of EC (2 or 10 mg/kg) in (N(G)-Nitro-L-arginine methyl ester) (L-NAME)-induced hypertensive rats had no effect on hypertension development, a weak effect on endothelial dysfunction induced by L-NAME and prevented the cardiac hypertrophy. By blocking nitric oxide (NO) production, EC suppressed the proatherogenic and proinflammatory status of the vascular wall (121). The second study was done in 11-deoxycorticosterone (DOCA)-salt-induced hypertensive rats and improvements in systolic blood pressure (SBP), proteinuria, and vascular dysfunction by the high dose of EC were recorded probably via oxidative stress suppression and nicotinamide adenine dinucleotide phosphate (NADPH) oxidase activity inhibition (122). One similar experiment about the effect of 28 days of low EC (1 mg/kg/day) in DOCAsalt-induced hypertensive rats showed the regulation of myocardial stiffness and left ventricular compliance, reduction of BP, and malondialdehyde (MDA) concentration with no effect on cardiac hypertrophy (123).

L-NAME hypertensive rats that were given GT ad libitum for 1 week showed a reduction in hypertension and improved arterial baroreceptor function through the mitigation of vascular and systemic oxidative stress (124). In a study of high fructose diet-fed rats which were supplemented with EC (20 mg/kg), BP increment was suppressed, oxidative stress was diminished and nitric oxide synthase (NOS) activity was boosted (125). 

One comparative study between low dose (0.2g/kg/day) and high dose (1 g/kg) of GT for 4 weeks in spontaneously hypertensive rats reported suppression of SBP increment and dose-dependent protection of left ventricular myocardium and aortic vascular smooth muscle cells from oxidative damages (126). 

A mechanistic study was designed to determine the involvement of miRNA in the anti-hypertensive effect of EGCG and it was found that miRNA-150-5p is responsible for this effect (127). Another study undertook several mechanisms such as anti-inflammatory and anti-oxidant pathways for GTE (2 or 4g/kg in diet) beneficial effects on NaCl-induced hypertensive rats (128).

An experiment by Wang *et al.* was done on the effects of EGCG on HTN-induced disorders. In addition to the anti-hypertensive effects, EGCG regulated impaired learning, memory, and locomotor activity, which is thought to be through anti-oxidative mechanisms (129).

In 2 studies by Yi *et al.* 4 weeks of bilateral PVN hypothalamic paraventricular nucleus (PVN) infusion of EGCG (20 µg/h) via osmotic minipumps in spontaneously hypertensive rats model and renovascular hypertensive rats model, resulted in anti-hypertensive effects which seemed to be through the suppression of inflammation and oxidative stress and led to regulation of neurotransmitters in PVN, helping to improve hypertension (130, 131). 

*Clinical studies*

There are a limited number of clinical studies about the effects of GT on hypertension.

A cross-sectional observational study that was done on 75 healthy individuals and 75 T2D patients showed remarkable improvements in SBP and diastolic blood pressure (DBP) after 90 days of GT consumption (112). SBP of 20 obese pre-hypertensive women was significantly reduced after supplementation with 500 mg GTE capsules 3 times daily for 4 weeks. DBP and other parameters showed no change in this crossover, randomized, double-blind, placebo-controlled clinical trial (132). 56 obese, hypertensive subjects who were given 1 GTE capsules daily for 3 months, experienced a remarkable reduction in SBP and DBP in a double-blind, placebo-controlled trial (25). Another experiment about the effects of GTE capsules (containing 75 mg EGCG, BD for 3 weeks) showed no effect on SBP, DBP and heart rate (HR) but a satisfactory effect on mean arterial BP (MAP) and rate pressure product (RPP) response 1 hour after an acute resistance exercise in 24 sedentary middle-aged women (133). Supplementation with GT (3 cups daily for 14 weeks) in pre-diabetic subjects caused suppression of MAP increment of both sexes in one randomized human cohort (120).

On the other hand, some experiments noted opposite effects on BP. For instance, in one observational study 76 women, who were given a GT decoction, a moderate elevation in BP, and a great reduction in HR have been reported (134). Moreover, in another controversial study in 29 older adults, drinking GT before lunch enhanced SBP and DBP significantly with no effect on HR. GT was introduced as a BP pressor in postprandial hypotension in the elderly (135).

**Table 1:** Effects of green tea and it’s components on dyslipidemia in animal studies

**Treatment features**	**Model**	**Effects**	**Reference**
Catechins and EGCG in functional drinks – 56 days	Rats fed high cholesterol and high sucrose diet	Cholesterol and LDL reduction	(73)
Catechins and EGCG in functional drinks	Rats fed high cholesterol and high sucrose diet	Improving serum anti-oxidant potential	(136)
GT ethanolic extract and powder – 8 weeks	Rats	Beneficial effects against hypercholesterolemia	(42)
GT 1% (w/v) in drinking water- last week of the 5-week study course	Rats fed a high cholesterol diet	COX-2 downregulation, oxidative DNA damage reduction, no significant effect on cholesterol level	(137)
GTE 50 mg/kg/day orally	HfrD fed rats	Decreasing myocardial fibrosis, increasing hepatic catalase activity	(138)
GT given as protective regimen (PG) and curative regimen (CG)	Rats fed a Hypercholesterolemic diet	Significant improvement of dyslipidemia, lower SOD activity in CG, stronger liver protection in PG, a significant decrease in Atherogenic Index	(139)
GTP in drinking water	Diet-induced obese rats	Reduced liver TG level	(62)
GTC	Diet-induced obese rat	Decreased liver TG levels	(68)
GTE 1 or 2 g/kg in a diet for 6 weeks	HFrD fed rats	Decreased TG levels	(103)
GT 500 mg/kg/day, 5days/week for 12 weeks	Diet-induced obese rat	Reduced liver and plasma lipid content, increased fatty acid oxidation	(70)
GTE	Diet-induced obese rat	Anti-lipidemia properties	(83)
GT 0.2 or 1 g/kg/day for 4 weeks	Spontaneously hypertensive rats	No effect on plasma total cholesterol	(126)
GT + GTC 30 or 100 mg	T2D rats	Decreased serum cholesterol, TG, LDL, vLDL	(140)
GTE 0.75% or 1.0% in diet	HFD fed rats born of obese dams	Decreased liver TG in offspring	(141)
GTP 200 mg/kg/day in drinking water for 6 weeks	HFrD fed rats	Attenuation of cholesterol, TG, and LDL	(104)
EGCG	Rabbits	Decreasing lipid deposition	(142)
EGCG oral and IP consecutive treatment for 14 days	Mice	Increasing serum lipids, hepatotoxicity (reversible)	(143)
EGCG	Diet-induced obese mice	Improved serum lipid profiles	(65)
GT added to the diet	Diet-induced obese mice	Reduced TG	(81)
EGCG as in situ hydrogel SC implant for 1 month	Diet-induced obese mice	Decreased total cholesterol, TG and LDL, increased HDL	(63)
EGCG 10, 20 and 40 mg/kg/day IP for 4 weeks	non-alcoholic fatty liver disease (NAFLD) mice	Modulate hyperlipidemia	(110)
EGCG supplementation for 12 weeks	senescence-accelerated mice (SAM) prone 8 (SAMP8)	Prevention of hepatic liver accumulation, modulate lipid homeostasis in skeletal muscles and liver	(98)
GTP 50,100 mg/kg for 20 days	Chicken	Reduced serum TG, cholesterol, and LDL levels suppressed fatty acid synthesis	(74)

GT: green tea; EGCG: Epigallocatechin gallate; GTE: green tea extract, GTP: green tea polyphenols; GTC: green tea catechin; HFD: high-fat diet; HfrD: high fructose diet; T2D: type-2 diabetes; IP: intraperitoneal; SC: subcutaneous; SOD: superoxide dismutase

**Table 2:** Effects of green tea and it’s components on obesity in animal studies

**Treatment features**	**Model**	**Effects**	**Reference**
FGT extract	Diet-induced obese mice	Reduced BW and fat mass with no effect on food intake, downregulation of lipogenic and inflammatory genes, modulation of gut microbiomes	(67)
EC in diet	Diet-induced obese mice	Downregulation of inflammation-related genes	(79)
EGCG in the diet for 17 weeks	Diet-induced obese mice	Reduced BW and inflammatory cytokines, increased fecal lipids	(38)
EGCG	Diet-induced obese mice	Reduced BW and fat infiltration in liver tissue, anti-inflammatory effects	(65)
EGCG- 10 mg/kg/day through gavage for 2 weeks	Diet-induced obese mice	Reduced fat mass, normalizing inflammatory and oxidative markers	(75)
GT added to the diet	Diet-induced obese mice	Reduction of adipose tissue, modulation of leptin level	(81)
EGCG for 17 weeks	Diet-induced obese mice	Reduced BW, liver and kidney weight	(69)
GTE 400 mg/kg through gavage for 8 weeks	Diet-induced obese mice	Reduced BW and adipose tissue and inflammatory cytokines, increased lipolysis	(71)
EGCG as in situ hydrogel SC implant for 1 month	Diet-induced obese mice	Modulation of weight gain	(63)
GT 2% in the diet	Diet-induced obese mice	Reduced BW, fat mass, liver weight, induced lipolysis, anti-inflammatory effects	(64)
EGCG oral administration for 30 days	Diet-induced obese mice	Inhibition of alpha-amylase activity reduced lipid accumulation	(82)
GTE in the diet for 8 weeks	Diet-induced obese mice	Reduced BW and fat mass, Modulation of PPAR-delta	(77)
EGCG 50 mg/kg/day for 10 weeks	Mice fed HFD	Decreased BW increment	(101)
GT 500 mg/kg for 12 weeks	HFD fed mice	Reduced BW, increased energy expenditure	(102)
GTE in diet in 3 regimens: GTE 1g/kg of diet for 3 days,GTE 1g/kg of diet for 28 days,GTE 0.1g/kg of diet for 28 days	Glutamate induced obese mice	No effect on average BW, decreased food intake in third regimen, reduced leptin level in the first regimen	(144)
GTE	Monosodium glutamate treated mice model	Decreased leptin levels	(80)
EGCG in drinking water for 3 days	Dextran sulfate sodium-treated mice model	Reduced BW, decreased protein and lipid digestion, anti-inflammatory effects	(66)
Green tea polyphenon E 0.1% in the diet for 7 weeks	db/db mice (leptin deficiency model)	Decreased mesenteric fat	(145)
GTE 2% in the diet for 8 weeks	RGS10 knockout mice fed a HFD	Suppressed HFD-induced obesity	(100)
EGCG 10, 20 and 40 mg/kg/day IP for 4 weeks	non-alcoholic fatty liver disease (NAFLD) mice	Reduced BW	(110)
GTC	Diet-induced obese rat	Modulation of BW and liver weight, modification of PPAR	(68)
GT 500 mg/kg/day, 5days/week for 12 weeks	Diet-induced obese rat	Reduced-fat synthesis, BW and fat depots, AMPK activation, modulation of metabolism-related genes	(70)
Green tea polyphenols in drinking water	Diet-induced obese rat	Reduced BW, Regulation of orexigenic, anorectic, and energy expenditure-related genes, modulation of anti-inflammation and anti-oxidant effects, estrogen-related actions	(62)
Green tea as functional drinks for 56 days	Diet-induced obese rat	Reduced BW	(73)
GTE	Diet-induced obese rat	Anti-obesity properties	(83)
GTE	Rat	Decreased BW, fat mass, and leptin levels, increased fecal lipids, and total daily energy consumption	(72)
GTE (2 or 4g/kg in diet)	NaCl-induced hypertensive rats	No effect on body mass	(128)
GTP 50,100 mg/kg for 20 days	Chicken	Reduced-fat masses, downregulation of lipid anabolism genes, and upregulation of lipid catabolism genes	(74)

BW: body weight; FGT: fermented green tea; EC: epicatechin; EGCG: epigallocatechin gallate; GT: green tea; GTE: green tea extract; GTC: green tea catechin; GTP: green tea polyphenols; SC: subcutaneous; HFD: high-fat diet; PPAR: Peroxisome Proliferator-Activated Receptor; AMPK: 5’ AMP-activated protein kinase; RGS10: Regulator of G-protein Signaling 10

**Table 3:** Effects of green tea and it’s components on diabetes in animal studies

**Treatment features**	**Model**	**Effects**	**Reference**
EGCG 10 mg/kg/day in drinking water GTE 10 mg/kg/day in drinking water	Mice administered 30% glucose	No significant effect on FBG, IPGTT, gAUC, insulin resistance, and HOMA-B, increasing insulin level	(146)
GTE in diet in 3 regimens: GTE 1g/kg of diet for 3 days,GTE 1g/kg of diet for 28 days,GTE 0.1g/kg of diet for 28 days	Glutamate induced obese mice	No effect on anti-oxidation systems, a significant reduction in insulin level	(144)
Green tea polyphenon E 0.1% in the diet for 7 weeks	db/db mice (leptin deficiency model)	Decreased FBG, increased insulin level	(145)
EC 20 mg/kg in the diet for 15 weeks	HFD-induced insulin resistance in mice	Improved insulin sensitivity	(97)
EC 2-20 mg/kg	HFD-induced insulin resistance in mice	Attenuation of insulin resistance	(107)
GTE 2% in the diet for 8 weeks	RGS10 knockout mice fed a HFD	Regulation of impaired glucose tolerance test and insulin resistance	(100)
EC 0.5% in drinking water	Non-obese diabetic mice	Increased plasma insulin level, decreased HbA1C concentrations	(109)
EGCG 0.05% in drinking water	Non-obese diabetic mice	Increased plasma insulin level, decreased HbA1C concentrations	(147)
EGCG 10, 20 and 40 mg/kg/day IP for 4 weeks	non-alcoholic fatty liver disease (NAFLD) mice	Mitigation of hyperglycemia, hyperinsulinemia, and insulin resistance in a dose-dependent manner	(110)
EGCG 50 mg/kg/day for 10 weeks	Mice fed HFD	Improved insulin sensitivity and glucose tolerance	(101)
EGCG supplementation for 12 weeks	senescence-accelerated mice (SAM) prone 8 (SAMP8)	Improved insulin sensitivity by attenuating BG and insulin level	(98)
GT 500 mg/kg for 12 weeks	HFD fed mice	Improved insulin sensitivity	(102)
EGCG 25 or 75 mg/kg i.p 3times/week for 17 weeks	HFD fed C57BL/6 mice	Remarkably reduced plasma glucose and insulin level	(69)
EC 200 mg/kg in diet	HFD fed C57BL/6 mice	Protection from insulin resistance	(79)
EGCG 1 or 10 μM in drinking water at embryonic day 5.5	Diabetic pregnant mice	EGCG 10 μM remarkably reduced neural tube defect incidence	(111)
FGT extract	Diet-induced obese mice	Decreased glucose intolerance	(67)
GT added to the diet	Diet-induced obese mice	Reduced BG and insulin levels	(81)
EGCG oral administration for 30 days	Diet-induced obese mice	Decreased serum glucose	(82)
GTE	Monosodium glutamate treated mice model	Decreased insulin levels	(80)
GTE 1 or 2 g/kg in the diet for 6 weeks	HFrD fed rats	Decreased glucose an insulin level, improved insulin resistance	(103)
Functional drinks containing catechins and EGCG	High cholesterol and high sucrose diet-fed rats	Mitigation of serum glucose and insulin levels	(73)
EGCG 3.2 g/kg in the diet for 16 weeks	HFD fed rats	Decreased fasting plasma insulin and homeostasis model assessment-insulin resistance index, increased glucose infusion rate	(148)
EC 20 mg/kg in the diet for 8 weeks	HFrD fed rats	Attenuation of insulin resistance	(96)
GT ad libitum for up to 90 days	T1D rats	Mitigation of periodontal breakdown and prevention of vascular disturbances	(149)
EGCG 50 mg/kg/day orally for 2 months	IDDM rats	Increased cardiac function synergistically with stem cell treatment	(150)
EGCG	Streptozotocin and HFD induced diabetic rats	Significant reduction in the expression and activity of P-glycoprotein	(151)
GT + GTC 30 or 100 mg	T2D rats	Decreased serum glucose level (more severe in GT+100 mg GTC)	(140)
GT in drinking water for 21 days	Streptozotocin-induced diabetic rats	Reduced hyperglycemia	(152)
GTE 200 mg/kg orally for 16 weeks	Streptozotocin-induced diabetic rats	Reduced BG and Hba1c level	(108)
GTE 0.75% or 1.0% in diet	HFD fed rats born of obese dams	Reduced insulin resistance in offspring	(141)
EGCG IV infusion with intralipid-heparin for 48 hours	Over-night fasted rats	Remarkably prohibited free fatty acid-induced peripheral insulin resistance	(153)
EGCG 2g/l as a beverage for 10 weeks	Streptozotocin-induced diabetic rats	Prevention of diabetes-induced loss of cavernous smooth muscle with no effect on vascular growth factor expression	(154)
GTP 200 mg/kg/day in drinking water for 6 weeks	HFrD fed rats	Mitigation of blood glucose and plasma insulin, improved insulin signaling	(104)
EGCG 25 mg/kg/day for 8 weeks	Streptozotocin-induced diabetic rats	Reduced glucose level	(105)
EGCG 1 or 10 mu mol/L	Day-9 rat conceptuses cultures	Attenuation of vasculopathy and malformations induced by hyperglycemia	(99)
Green tea ethanolic extract and powder – 8 weeks	Hyperglycemic rats	Reduced serum glucose level	(42)
GTP in drinking water	Diet-induced obese rat	Reduced BG, insulin resistance	(62)
GT 500 mg/kg/day, 5days/week for 12 weeks	Diet-induced obese rat	Restpred insulin sensitivity	(70)
GTC 150 or 300 mg/kg/day in the diet for 4 weeks GTC 20 mg/kg/day in diet for 45 days	HFD fed KK-ay and C57BL/6 mice HFD fed rats	Attenuated glucose level and enhanced glucose tolerance	(106)

FGT: fermented green tea; EC: epicatechin; EGCG: epigallocatechin gallate; GT: green tea; GTE: green tea extract; GTP: green tea polyphenols; GTC: green tea catechin; HFD: high-fat diet; RGS10: Regulator of G-protein Signaling 10; HFrD: high fructose diet; T1D: type-1 diabetes; T2D: type-2 diabetes; IDDM: Insulin-dependent diabetes mellitus; BG: blood glucose; FBG: fasting blood glucose; HOMA: homeostatic model assessment; IPGTT: intraperitoneal glucose tolerance test; gAUC: glucose area under the curve; Hba1c: glycosylated hemoglobin A1c

## Conclusion

In the present study, different experiments about the effects and probable mechanisms of GT and its catechins on MetS parameters such as dyslipidemia, obesity, diabetes, and hypertension were reviewed. The majority of studies in animal and clinical studies agreed on the affirmative effects of GT. However, there were contradictory experiments in some topics that need further studies for clarification. Almost all of the experiments were suggestive of the beneficial effects of GT on MetS parameters. 

Animal studies and the scant number of clinical experiments are in good agreement about the beneficial effects of GT on dyslipidemia and atherogenesis through anti-oxidative and anti-inflammatory properties. The anti-obesity effects of GT were assessed in most of the animal and clinical experiments. The prevalent mechanisms are as follows: suppressing inflammation, oxidative stress, and leptin levels, stimulating lipolysis, and regulating Peroxisome Proliferator-Activated Receptor (PPAR). The majority of animal studies agree on the valuable effects of GT on diabetes by affecting diabetes modulators, anti-inflammatory, and anti-oxidative properties. However, the clinical experiments were not in total agreement and needed more investigation. Almost all of the animal studies showed positive effects of GT on hypertension, mostly through oxidative stress and inflammation suppression. However, the clinical studies were limited and controversial. Hence there is a need for more studies to reach a final outcome. 

## Authors’ Contributions

EE Preparation of original draft; BMR Critical revision of the paper, supervision of the research; HH Study conception, design and supervision of the research. All authors have agreed to the contents and approved the final version for publication

## Conflicts of Interest

The authors declare that there are no conflicts of interest.

## References

[1] McCracken E, Monaghan M, Sreenivasan S (2018). Pathophysiology of the metabolic syndrome. Clin Dermatol.

[2] Reaven GM (1988). Role of insulin resistance in human disease. Diabetes.

[3] Grundy SM (2016). Metabolic syndrome update. Trends in Cardiovascular Medicine.

[4] Akaberi M, Hosseinzadeh H (2016). Grapes (Vitis vinifera) as a potential candidate for the therapy of the metabolic syndrome. Phytother Res.

[5] Dehghani S, Mehri S, Hosseinzadeh H (2019). The effects of Crataegus pinnatifida (Chinese hawthorn) on metabolic syndrome: A review. Iran J Basic Med Sci.

[6] Hassani FV, Shirani K, Hosseinzadeh H (2016). Rosemary (Rosmarinus officinalis) as a potential therapeutic plant in metabolic syndrome: A review. Naunyn Schmiedebergs Arch Pharmacol.

[7] Hosseini A, Hosseinzadeh H (2015). A review on the effects of Allium sativum (Garlic) in metabolic syndrome. J Endocrinol Invest.

[8] Razavi BM, Hosseinzadeh H (2014). A review of the effects of Nigella sativa L and its constituent, thymoquinone, in metabolic syndrome. J Endocrinol Invest.

[9] Razavi BM, Hosseinzadeh H, Watson RR, Preedy VR (543). Chapter 34 - a review of the effects of Citrus paradisi (grapefruit) and its flavonoids, naringin, and naringenin in metabolic syndrome. Bioactive food as dietary interventions for diabetes (second edition): Academic Press; 2019.

[10] Sanati S, Razavi BM, Hosseinzadeh H (2018). A review of the effects of Capsicum annuum L and its constituent, capsaicin, in metabolic syndrome. Iran J Basic Med Sci.

[11] Shakib Z, Shahraki N, Razavi BM, Hosseinzadeh H (2019). Aloe vera as an herbal medicine in the treatment of metabolic syndrome: A review. Phytother Res.

[12] Tabeshpour J, Imenshahidi M, Hosseinzadeh H (2017). A review of the effects of Berberis vulgaris and its major component, berberine, in metabolic syndrome. Iran J Basic Med Sci.

[13] Tabeshpour J, Razavi BM, Hosseinzadeh H (2017). Effects of avocado (Persea americana) on metabolic syndrome: A comprehensive systematic review. Phytother Res.

[14] Tajmohammadi A, Razavi BM, Hosseinzadeh H (2018). Silybum marianum (milk thistle) and its main constituent, silymarin, as a potential therapeutic plant in metabolic syndrome: A review. Phytother Res.

[15] Tousian Shandiz H, Razavi BM, Hosseinzadeh H (2017). Review of Garcinia mangostana and its xanthones in metabolic syndrome and related complications. Phytother Res.

[16] Razavi BM, Hosseinzadeh H (2017). Saffron: A promising natural medicine in the treatment of metabolic syndrome. J Sci Food Agric.

[17] Mollazadeh H, Hosseinzadeh H (2016). Cinnamon effects on metabolic syndrome: a review based on its mechanisms. Iran J Basic Med Sci.

[18] Hosseinzadeh H, Nassiri-Asl M (2014). Review of the protective effects of rutin on the metabolic function as an important dietary flavonoid. J Endocrinol Invest.

[19] Alappat B, Sarna JA, Truong C (2015). Anticancer and anti-oxidant properties of flavored green tea extracts. J Agric Life Sci.

[20] Chacko SM, Thambi PT, Kuttan R, Nishigaki I (2010). Beneficial effects of green tea: A literature review. Chin Med.

[21] Afzal M, Safer AM, Menon M (2015). Green tea polyphenols and their potential role in health and disease. Inflammopharmacology.

[22] Ohishi T, Goto S, Monira P, Isemura M, Nakamura Y (2016). Anti-inflammatory action of green tea. Antiinflamm Antiallergy Agents Med Chem.

[23] Maiti S, Nazmeen A, Medda N, Patra R, Ghosh TK (2019). Flavonoids green tea against oxidant stress and inflammation with related human diseases. Clin Nutr Exp.

[24] Huang J, Wang Y, Xie Z, Zhou Y, Zhang Y, Wan X (2014). The anti-obesity effects of green tea in human intervention and basic molecular studies. Eur J Clin Nutr.

[25] Bogdanski P, Suliburska J, Szulinska M, Stepien M, Pupek-Musialik D, Jablecka A (2012). Green tea extract reduces blood pressure, inflammatory biomarkers, and oxidative stress and improves parameters associated with insulin resistance in obese, hypertensive patients. Nutr Res.

[26] Batista Gde A, Cunha CL, Scartezini M, von der Heyde R, Bitencourt MG, Melo SF (2009). Prospective double-blind crossover study of Camellia sinensis (green tea) in dyslipidemias. Arq Bras Cardiol.

[27] Bornhoeft J, Castaneda D, Nemoseck T, Wang P, Henning SM, Hong MY (2012). The protective effects of green tea polyphenols: lipid profile, inflammation, and antioxidant capacity in rats fed an atherogenic diet and dextran sodium sulfate. J Med Food.

[28] Miyata Y, Shida Y, Hakariya T, Sakai H (2019). Anti-Cancer effects of green tea polyphenols against prostate cancer. Molecules.

[29] Butt MS, Ahmad RS, Sultan MT, Qayyum MM, Naz A (2015). Green tea and anticancer perspectives: Updates from last decade. Crit Rev Food Sci Nutr.

[30] Pervin M, Unno K, Ohishi T, Tanabe H, Miyoshi N, Nakamura Y (2018). Beneficial effects of green tea catechins on neurodegenerative diseases. Molecules.

[31] Esmaeelpanah E, Razavi BM, Vahdati Hasani F, Hosseinzadeh H (2018). Evaluation of epigallocatechin gallate and epicatechin gallate effects on acrylamide-induced neurotoxicity in rats and cytotoxicity in PC 12 cells. Drug Chem Toxicol.

[32] Esmaeelpanah E, Rahmatkhah A, Poormahmood N, Razavi BM, Vahdati Hassani F, Hosseinzadeh H (2015). Protective effect of green tea aqueous extract on acrylamide induced neurotoxicity. Jundishapur J Nat Pharm Prod.

[33] Fazly Bazzaz BS, Sarabandi S, Khameneh B, Hosseinzadeh H (2016). Effect of catechins, green tea extract and methylxanthines in combination with gentamicin against Staphylococcus aureus and Pseudomonas aeruginosa: - Combination therapy against resistant bacteria. J pharmacopuncture.

[34] Rameshrad M, Razavi BM, Hosseinzadeh H (2017). Protective effects of green tea and its main constituents against natural and chemical toxins: A comprehensive review. Food Chem Toxicol.

[35] Saeki K, Hayakawa S, Nakano S, Ito S, Oishi Y, Suzuki Y (2018). In vitro and in silico studies of the molecular interactions of epigallocatechin-3-o-gallate (egcg) with proteins that explain the health benefits of green tea. Molecules.

[36] Saboya PP, Bodanese LC, Zimmermann PR, Gustavo AdS, Assumpção CM, Londero F (2016). Metabolic syndrome and quality of life: A systematic review. Revista latino-americana de enfermagem.

[37] Razavi BM, Lookian F, Hosseinzadeh H (2017). Protective effects of green tea on olanzapine-induced-metabolic syndrome in rats. Biomed Pharmacother.

[38] Chen YK, Cheung C, Reuhl KR, Liu AB, Lee MJ, Lu YP (2011). Effects of green tea polyphenol (-)-epigallocatechin-3-gallate on newly developed high-fat/Western-style diet-induced obesity and metabolic syndrome in mice. J Agric Food Chem.

[39] Nugroho DA, Lukitasari M, Rohman MS (2017). Green tea extract improved blood pressure and metabolic profile of metabolic syndrome rat model. J Hypertens.

[40] Nugroho DA, Lukitasari M, Rohman MS (2018). Dose-dependent effects of green tea extract on adiponectin level and adiponectin receptor gene expression in metabolic syndrome rat models. J Hypertens.

[41] Lukitasari M, Nugroho DA, Rohman MS (2018). Green tea extract administration had a beneficial effect on ppar alpha and ppar gamma gene expression in metabolic syndrome rat model. J Hypertens.

[42] Yousaf S, Butt MS, Suleria HA, Iqbal MJ (2014). The role of green tea extract and powder in mitigating metabolic syndromes with special reference to hyperglycemia and hypercholesterolemia. Food Funct.

[43] Basu A, Du M, Sanchez K, Leyva MJ, Betts NM, Blevins S (2011). Green tea minimally affects biomarkers of inflammation in obese subjects with metabolic syndrome. Nutrition.

[44] Basu A, Sanchez K, Leyva MJ, Wu MY, Betts NM, Aston CE (2010). Green tea supplementation affects body weight, lipids, and lipid peroxidation in obese subjects with metabolic syndrome. J Am Coll Nutr.

[45] Mortazavi F, Paknahad Z, Hasanzadeh A (2019). Effect of green tea consumption on the metabolic syndrome indices in women: A clinical trial study. Nutr Food Sci.

[46] Vieira Senger AE, Schwanke CH, Gomes I, Valle Gottlieb MG (2012). Effect of green tea (Camellia sinensis) consumption on the components of metabolic syndrome in elderly. J Nutr Health Aging.

[47] Basu A, Betts NM, Mulugeta A, Tong C, Newman E, Lyons TJ (2013). Green tea supplementation increases glutathione and plasma antioxidant capacity in adults with the metabolic syndrome. Nutr Res.

[48] Kim E, Lee M, Kim SS, Kim JH, Jeon YK, Kim BH (2016). Green tea but not coffee consumption is inversely associated with metabolic syndrome; An epidemiological study in Korean adults. Diabetes Res Clin Prac.

[49] Morrison M, van der Heijden R, Heeringa P, Kaijzel E, Verschuren L, Blomhoff R (2014). Epicatechin attenuates atherosclerosis and exerts anti-inflammatory effects on diet induced human-crp and nfkb in vivo. Atherosclerosis.

[50] Ramesh E, Jayakumar T, Elanchezhian R, Sakthivel M, Geraldine P, Thomas PA (2009). Green tea catechins, alleviate hepatic lipidemic-oxidative injury in Wistar rats fed an atherogenic diet. Chem Biol Interact.

[51] Ramesh E, Geraldine P, Thomas PA (2010). Regulatory effect of epigallocatechin gallate on the expression of C-reactive protein and other inflammatory markers in an experimental model of atherosclerosis. Chem Biol Interact.

[52] Miltonprabu S, Thangapandiyan S (2015). Epigallocatechin gallate potentially attenuates Fluoride induced oxidative stress mediated cardiotoxicity and dyslipidemia in rats. J Trace Elem Med Biol.

[53] Wang W, Zhang ZZ, Wu Y, Wang RQ, Chen JW, Chen J (2018). (-)-Epigallocatechin-3-Gallate ameliorates atherosclerosis and modulates hepatic lipid metabolic gene expression in apolipoprotein e knockout mice: Involvement of TTC39B. Front Pharmacol.

[54] Cai Y, Kurita-Ochiai T, Hashizume T, Yamamoto M (2013). Green tea epigallocatechin-3-gallate attenuates Porphyromonas gingivalis-induced atherosclerosis. Pathog Dis.

[55] Ding S, Jiang J, Yu P, Zhang G, Zhang G, Liu X (2017). Green tea polyphenol treatment attenuates atherosclerosis in high-fat diet-fed apolipoprotein E-knockout mice via alleviating dyslipidemia and up-regulating autophagy. PLoS One.

[56] Minatti J, Wazlawik E, Hort MA, Zaleski FL, Ribeiro-do-Valle RM, Maraschin M (2012). Green tea extract reverses endothelial dysfunction and reduces atherosclerosis progression in homozygous knockout low-density lipoprotein receptor mice. Nutr Res.

[57] Gutierrez-Salmean G, Meaney E, Lanaspa MA, Cicerchi C, Johnson RJ, Dugar S (2016). A randomized, placebo-controlled, double-blind study on the effects of (-)-epicatechin on the triglyceride/HDLc ratio and cardiometabolic profile of subjects with hypertriglyceridemia: Unique in vitro effects. Int J Cardiol.

[58] Gutiérrez-Salmeán G, Ortiz-Vilchis P, Vacaseydel CM, Rubio-Gayosso I, Meaney E, Villarreal F (2014). Acute effects of an oral supplement of (-)-epicatechin on postprandial fat and carbohydrate metabolism in normal and overweight subjects. Food Func.

[59] Huang LH, Liu CY, Wang LY, Huang CJ, Hsu CH (2018). Effects of green tea extract on overweight and obese women with high levels of low density-lipoprotein-cholesterol (LDL-C): a randomised, double-blind, and cross-over placebo-controlled clinical trial. BMC Complement Altern Med.

[60] Alves Ferreira M, Oliveira Gomes AP, Guimaraes de Moraes AP, Ferreira Stringhini ML, Mota JF, Siqueira Guedes Coelho A (2017). Green tea extract outperforms metformin in lipid profile and glycaemic control in overweight women: A double-blind, placebo-controlled, randomized trial. Clin Nutr ESPEN.

[61] Liu CY, Huang CJ, Huang LH, Chen IJ, Chiu JP, Hsu CH (2014). Effects of green tea extract on insulin resistance and glucagon-like peptide 1 in patients with type 2 diabetes and lipid abnormalities: A randomized, double-blinded, and placebo-controlled trial. Plos One.

[62] Lu CW, Zhu WB, Shen CL, Gao WM (2012). Green tea polyphenols reduce body weight in rats by modulating obesity-related genes. Plos One.

[63] Zhang XZ, Guan J, Cai SL, Du Q, Guo ML (2015). Polymeric in situ hydrogel implant of epigallocatechin gallate (EGCG) for prolonged and improved antihyperlipidemic and anti-obesity activity: Preparation and characterization. J Biomater Tissue Eng.

[64] Cichello SA, Begg DP, Jois M, Weisinger RS (2013). Prevention of diet-induced obesity in C57BL/BJ mice with addition of 2 % dietary green tea but not with cocoa or coffee to a high-fat diet. Med J Nutrition Metab.

[65] Byun JK, Yoon BY, Jhun JY, Oh HJ, Kim EK, Min JK (2014). Epigallocatechin-3-gallate ameliorates both obesity and autoinflammatory arthritis aggravated by obesity by altering the balance among CD4(+) T-cell subsets. Immunol Lett.

[66] Bitzer ZT, Elias RJ, Vijay-Kumar M, Lambert JD (2016). (-)-Epigallocatechin-3-gallate decreases colonic inflammation and permeability in a mouse model of colitis, but reduces macronutrient digestion and exacerbates weight loss. Mol Nutr Food Res.

[67] Seo DB, Jeong HW, Cho D, Lee BJ, Lee JH, Choi JY (2015). Fermented green tea extract alleviates obesity and related complications and alters gut microbiota composition in diet-induced obese mice. J Med Food.

[68] Yan J, Zhao Y, Zhao B (2013). Green tea catechins prevent obesity through modulation of peroxisome proliferator-activated receptors. Sci China Life Sci.

[69] Sampath C, Rashid MR, Sang S, Ahmedna M (2017). Green tea epigallocatechin 3-gallate alleviates hyperglycemia and reduces advanced glycation end products via nrf2 pathway in mice with high fat diet-induced obesity. Biomed Pharmacother.

[70] Rocha A, Bolin AP, Cardoso CA, Otton R (2016). Green tea extract activates AMPK and ameliorates white adipose tissue metabolic dysfunction induced by obesity. Eur J Nutr.

[71] A Cunha C, Lira F, Rosa J, Pimentel G, Souza G, Silva C (2013). Green tea extract supplementation induces the lipolytic pathway, attenuates obesity, and reduces low-grade inflammation in mice fed a high-fat diet. Mediators Inflamm.

[72] Sogawa M, Seura T, Kohno S, Hirasaka K, Yamaguchi Y, Takagaki R (2009). Awa (Tokushima) lactate-fermented tea as well as green tea enhance the effect of diet restriction on obesity in rats. J Med Invest.

[73] Ahmad RS, Butt MS, Sultan MT, Mushtaq Z, Ahmad S, Dewanjee S (2015). Preventive role of green tea catechins from obesity and related disorders especially hypercholesterolemia and hyperglycemia. J Transl Med.

[74] Huang JB, Zhang Y, Zhou YB, Zhang ZZ, Xie ZW, Zhang JS (2013). Green tea polyphenols alleviate obesity in broiler chickens through the regulation of lipid-metabolism-related genes and transcription factor expression. J Agric Food Chem.

[75] Andre DM, Horimoto CM, Calixto MC, Alexandre EC, Antunes E (2018). Epigallocatechin-3-gallate protects against the exacerbation of allergic eosinophilic inflammation associated with obesity in mice. Int Immunopharmacol.

[76] Guo XJ, Cheng M, Zhang X, Cao JX, Wu ZF, Weng PF (2017). Green tea polyphenols reduce obesity in high-fat diet-induced mice by modulating intestinal microbiota composition. Int J Food Sci Technol.

[77] Yamashita S, Hirashima A, Lin IC, Bae J, Nakahara K, Murata M (2018). Saturated fatty acid attenuates anti-obesity effect of green tea. Sci Rep.

[78] Ueda M, Ashida H (2012). Green tea prevents obesity by increasing expression of insulin-like growth factor binding protein-1 in adipose tissue of high-fat diet-fed mice. J Agric Food Chem.

[79] Sano T, Nagayasu S, Suzuki S, Iwashita M, Yamashita A, Shinjo T (2017). Epicatechin downregulates adipose tissue CCL19 expression and thereby ameliorates diet-induced obesity and insulin resistance. Nutr Metab Cardiovasc Dis.

[80] Bousova I, Matouskova P, Bartikova H, Szotakova B, Hanusova V, Tomankova V (2016). Influence of diet supplementation with green tea extract on drug-metabolizing enzymes in a mouse model of monosodium glutamate-induced obesity. Eur J Nutr.

[81] Lee LS, Choi JH, Sung MJ, Hur JY, Hur HJ, Park JD (2015). Green tea changes serum and liver metabolomic profiles in mice with high-fat diet-induced obesity. Mol Nutr Food Res.

[82] Zhan W, Liu Y, Li DP, Liu Y (2016). Advancing insights on the anti-obesity biochemical mechanism of (-)-epigallocatechin gallate (EGCG) by inhibiting alpha-amylase activity. Rsc Advances.

[83] Thomas J, Thomas G (2013). Effect of catechin rich green tea (Camellia Sinensis) extracts on obesity triggered hepatic steatosis in rats fed with HFCS. Int J Pharma Bio Sci.

[84] Nabi BN, Sedighinejad A, Haghighi M, Farzi F, Rimaz S, Atrkarroushan Z (2018). The anti-obesity effects of green tea: A controlled, randomized, clinical trial. Iran Red Crescent Med J.

[85] Levy Y, Narotzki B, Reznick AZ (2017). Green tea, weight loss and physical activity. Clin Nutr.

[86] Tsai Ch H, Chiu WC, Yang NC, Ouyang CM, Yen YH (2009). A novel green tea meal replacement formula for weight loss among obese individuals: a randomized controlled clinical trial. Int J Food Sci Nutr.

[87] Gholamreza S, Reza H, Esmailzade A, Alireza BM, Nodoushan IS, Hajian N (2013). Comparison between aerobic exercise and consumption of green tea on weight loss in overweighted men. Sport Sci.

[88] Thielecke F, Rahn G, Bohnke J, Adams F, Birkenfeld A, Jordan J (2010). Epigallocatechin-3-gallate and postprandial fat oxidation in overweight/obese male volunteers: a pilot study. Eur J Clin Nutr.

[89] Josic J, Olsson AT, Wickeberg J, Lindstedt S, Hlebowicz J (2010). Does green tea affect postprandial glucose, insulin and satiety in healthy subjects: A randomized controlled trial. Nutr J.

[90] Mohammadi S, Hosseinzadeh Attar MJ, Karimi M, Hosseinnezhad A, Eshraghian MR, Hosseini SH (2010). The effects of green tea extract on serum adiponectin concentration and insulin resistance in patients with type 2 diabetes mellitus. J Zanjan Univ Med Sci Health Services.

[91] Mousavi A, Vafa M, Neyestani T, Khamseh M, Hoseini F (2013). The effects of green tea consumption on metabolic and anthropometric indices in patients with Type 2 diabetes. J Res Med Sci.

[92] Tehrani HG, Allahdadian M, Zarre F, Ranjbar H, Allahdadian F (2017). Effect of green tea on metabolic and hormonal aspect of polycystic ovarian syndrome in overweight and obese women suffering from polycystic ovarian syndrome: A clinical trial. J Educ Health Promot.

[93] Mielgo-Ayuso J, Barrenechea L, Alcorta P, Larrarte E, Margareto J, Labayen I (2014). Effects of dietary supplementation with epigallocatechin-3-gallate on weight loss, energy homeostasis, cardiometabolic risk factors and liver function in obese women: randomised, double-blind, placebo-controlled clinical trial. Br J Nutr.

[94] Soori R, Safei A, Pournemati P, Ghram A (2018). Green tea consumption reduces apelin and orexin-A in overweight and obese women with different training modalities. Sport Sci Health.

[95] Balsan G, Pellanda LC, Sausen G, Galarraga T, Zaffari D, Pontin B (2019). Effect of yerba mate and green tea on paraoxonase and leptin levels in patients affected by overweight or obesity and dyslipidemia: a randomized clinical trial. Nutr J.

[96] Bettaieb A, Vazquez Prieto MA, Rodriguez Lanzi C, Miatello RM, Haj FG, Fraga CG (2014). (-)-Epicatechin mitigates high-fructose-associated insulin resistance by modulating redox signaling and endoplasmic reticulum stress. Free Radic Biol Med.

[97] Cremonini E, Bettaieb A, Haj FG, Fraga CG, Oteiza PI (2016). (-)-Epicatechin improves insulin sensitivity in high fat diet-fed mice. Arch Biochem Biophys.

[98] Liu HW, Chan YC, Wang MF, Wei CC, Chang SJ (2015). Dietary (-)-Epigallocatechin-3-gallate supplementation counteracts aging-associated skeletal muscle insulin resistance and fatty liver in senescence-accelerated mouse. J Agric Food Chem.

[99] Yang PX, Li H (2010). Epigallocatechin-3-gallate ameliorates hyperglycemia-induced embryonic vasculopathy and malformation by inhibition of Foxo3a activation. Am J Obstet Gynecol.

[100] Fang X, Chung J, Olsen E, Snider I, Earls RH, Jeon J (2019). Depletion of regulator-of-G-protein signaling-10 in mice exaggerates high-fat diet-induced insulin resistance and inflammation, and this effect is mitigated by dietary green tea extract. Nutr Res.

[101] Jang HJ, Ridgeway SD, Kim JA (2013). Effects of the green tea polyphenol epigallocatechin-3-gallate on high-fat diet-induced insulin resistance and endothelial dysfunction. Am J Physiol Endocrinol Metab.

[102] Otton R, Bolin AP, Ferreira LT, Marinovic MP, Rocha ALS, Mori MA (2018). Polyphenol-rich green tea extract improves adipose tissue metabolism by down-regulating miR-335 expression and mitigating insulin resistance and inflammation. J Nutr Biochem.

[103] Hininger-Favier I, Benaraba R, Coves S, Anderson RA, Roussel AM (2009). Green tea extract decreases oxidative stress and improves insulin sensitivity in an animal model of insulin resistance, the fructose-fed rat. J Am Coll Nutr.

[104] Qin BL, Polansky MM, Harry D, Anderson RA (2010). Green tea polyphenols improve cardiac muscle mRNA and protein levels of signal pathways related to insulin and lipid metabolism and inflammation in insulin-resistant rats. Mol Nutr Food Res.

[105] Roghani M, Baluchnejadmojarad T (2010). Hypoglycemic and hypolipidemic effect and antioxidant activity of chronic epigallocatechin-gallate in streptozotocin-diabetic rats. Pathophysiology.

[106] Yan J, Zhao Y, Suo S, Liu Y, Zhao B (2012). Green tea catechins ameliorate adipose insulin resistance by improving oxidative stress. Free Radic Biol Med.

[107] Cremonini E, Wang ZW, Bettaieb A, Adamo AM, Daveri E, Mills DA (2018). (-)-Epicatechin protects the intestinal barrier from high fat diet-induced permeabilization: Implications for steatosis and insulin resistance. Redox Biol.

[108] Kumar B, Gupta SK, Nag TC, Srivastava S, Saxena R (2012). Green tea prevents hyperglycemia-induced retinal oxidative stress and inflammation in streptozotocin-induced diabetic rats. Ophthalmic Res.

[109] Fu Z, Yuskavage J, Liu DM (2013). Dietary flavonol epicatechin prevents the onset of type 1 diabetes in nonobese diabetic mice. J Agric Food Chem.

[110] Gan L, Meng ZJ, Xiong RB, Guo JQ, Lu XC, Zheng ZW (2015). Green tea polyphenol epigallocatechin-3-gallate ameliorates insulin resistance in non-alcoholic fatty liver disease mice. Acta Pharmacol Sin.

[111] Zhong J, Xu C, Reece EA, Yang P (2016). The green tea polyphenol EGCG alleviates maternal diabetes–induced neural tube defects by inhibiting DNA hypermethylation. Am J Obstet Gynecol.

[112] Shah T, Shaikh F, Ansari S (2017). To determine the effects of green tea on blood pressure of healthy and type 2 diabetes mellitus (DM) individuals. J Liaquat Univ Med Health Sci.

[113] Lahirin R, Permadhi I, Mudjihartini N, Rahmawati R, Sugianto R (2015). Additional benefit of higher dose green tea in lowering postprandial blood glucose. Med J Indonesia.

[114] Maruyama K, Iso H, Sasaki S, Fukino Y (2009). The association between concentrations of green tea and blood glucose levels. J Clin Biochem Nutr.

[115] Zhang H, Su S, Yu X, Li Y (2017). Dietary epigallocatechin 3-gallate supplement improves maternal and neonatal treatment outcome of gestational diabetes mellitus: a double-blind randomised controlled trial. J Hum Nutr Diet.

[116] Martin BJ, MacInnis MJ, Gillen JB, Skelly LE, Gibala MJ (2016). Short-term green tea extract supplementation attenuates the postprandial blood glucose and insulin response following exercise in overweight men. Appl Physiol Nutr Metab.

[117] Huang SM, Chang YH, Chao YC, Lin JA, Wu CH, Lai CY (2013). EGCG-rich green tea extract stimulates sRAGE secretion to inhibit S100A12-RAGE axis through ADAM10-mediated ectodomain shedding of extracellular RAGE in type 2 diabetes. Mol Nutr Food Res.

[118] Brown AL, Lane J, Coverly J, Stocks J, Jackson S, Stephen A (2009). Effects of dietary supplementation with the green tea polyphenol epigallocatechin-3-gallate on insulin resistance and associated metabolic risk factors: Randomized controlled trial. Br J Nutr.

[119] Liu XN, Xu WH, Cai H, Gao YT, Li HL, Ji BT (2018). Green tea consumption and risk of type 2 diabetes in Chinese adults: the Shanghai women’s health study and the shanghai men’s health study. Int J Epidemiol.

[120] Toolsee NA, Aruoma OI, Gunness TK, Kowlessur S, Dambala V, Murad F (2013). Effectiveness of green tea in a randomized human cohort: Relevance to diabetes and its complications. Biomed Res Int.

[121] Gomez-Guzman M, Jimenez R, Sanchez M, Romero M, O’Valle F, Lopez-Sepulveda R (2011). Chronic (-)-epicatechin improves vascular oxidative and inflammatory status but not hypertension in chronic nitric oxide-deficient rats. Br J Nutr.

[122] Gomez-Guzman M, Jimenez R, Sanchez M, Zarzuelo MJ, Galindo P, Quintela AM (2012). Epicatechin lowers blood pressure, restores endothelial function, and decreases oxidative stress and endothelin-1 and NADPH oxidase activity in DOCA-salt hypertension. Free Radic Biol Med.

[123] Jackson D, Connolly K, Batacan R, Ryan K, Vella R, Fenning A (2018). (-)-Epicatechin reduces blood pressure and improves left ventricular function and compliance in deoxycorticosterone acetate-salt hypertensive rats. Molecules.

[124] Garcia ML, Pontes RB, Nishi EE, Ibuki FK, Oliveira V, Sawaya ACH (2017). The antioxidant effects of green tea reduces blood pressure and sympathoexcitation in an experimental model of hypertension. J Hypertens.

[125] Litterio MC, Prieto MAV, Adamo AM, Elesgaray R, Oteiza PI, Galleano M (2015). (-)-Epicatechin reduces blood pressure increase in high-fructose-fed rats: effects on the determinants of nitric oxide bioavailability. J Nutr Biochem.

[126] Liang YR, Ma SC, Luo XY, Xu JY, Wu MY, Luo YW (2011). Effects of green tea on blood pressure and hypertension-induced cardiovascular damage in spontaneously hypertensive rat. Food Sci Biotechnol.

[127] Qian BJ, Tian CC, Ling XH, Yu LL, Ding FY, Huo JH (2018). miRNA-150-5p associate with antihypertensive effect of epigallocatechin-3-gallate revealed by aorta miRNome analysis of spontaneously hypertensive rat. Life Sci.

[128] Szulinska M, Stepien M, Kregielska-Narozna M, Suliburska J, Skrypnik D, Bak-Sosnowska M (2017). Effects of green tea supplementation on inflammation markers, antioxidant status and blood pressure in NaCl-induced hypertensive rat model. Food Nutr Res.

[129] Wang MH, Chang WJ, Soung HS, Chang KC (2012). (-)-Epigallocatechin-3-gallate decreases the impairment in learning and memory in spontaneous hypertension rats. Behav Pharmacol.

[130] Yi QY, Li HB, Qi J, Yu XJ, Huo CJ, Li X (2016). Chronic infusion of epigallocatechin-3-O-gallate into the hypothalamic paraventricular nucleus attenuates hypertension and sympathoexcitation by restoring neurotransmitters and cytokines. Toxicol Lett.

[131] Yi QY, Qi J, Yu XJ, Li HB, Zhang Y, Su Q (2016). Paraventricular nucleus infusion of epigallocatechin-3-O-gallate improves renovascular hypertension. Cardiovasc Toxicol.

[132] Nogueira LP, Nogueira Neto JF, Klein MR, Sanjuliani AF (2017). Short-term effects of green tea on blood pressure, endothelial function, and metabolic profile in obese prehypertensive women: A crossover randomized clinical trial. J Am Coll Nutr.

[133] Arazi H, Samami N, Kheirkhah J, Taati B (2014). The effect of three weeks green tea extract consumption on blood pressure, heart rate responses to a single bout resistance exercise in hypertensive women. High Blood Press Cardiovasc Prev.

[134] Ullah N, Rafique N, Nazir A, Anwar S, Altaf N, Ahmed G (2011). Effect of decoction of Camellia sinensis on blood pressure and heart rate. Medical Forum Mon.

[135] Son JT, Lee E (2012). Effects of green tea ingestion on postprandial drops in blood pressure in older adults. J Gerontol Nurs.

[136] Ahmad RS, Butt MS, Huma N, Sultan MT (2013). Green tea catechins based functional drink (Green cool) improves the antioxidant status of SD rats fed on high cholesterol and sucrose diets. Pak J Pharm Sci.

[137] de Moraes BB, Pasquini G, Aguiar O, Gollucke APB, Ihara SSM, Tenorio NM (2011). Protective effects of green tea against hepatic injury induced by high-cholesterol diet in rats: histopathological analysis, oxidative DNA damage and COX-2 expression. Hepatol Int.

[138] Jung MH, Seong PN, Kim MH, Myong NH, Chang MJ (2013). Effect of green tea extract microencapsulation on hypertriglyceridemia and cardiovascular tissues in high fructose-fed rats. Nutr Res Pract.

[139] Labdi A, Amiali M, Bachir YN, Merouane A, Dahman-Zouambi A, Koceir EA (2018). Green tea extract attenuates non alcoholic fatty liver disease by decreasing hyperlipidemia and enhancing Superoxide dismutase activity in cholesterol-fed rats. Med J Nutrition Metab.

[140] El-Sayed Mostafa U (2014). Effect of green tea and green tea rich with catechin on blood glucose levels, serum lipid profile and liver and kidney functions in diabetic rats. Jordan J Biol Sci.

[141] Li SY, Tse IMY, Li ETS (2012). Maternal green tea extract supplementation to rats fed a high-fat diet ameliorates insulin resistance in adult male offspring. J Nutr Biochem.

[142] Hong ZY, Xu YQ, Yin JF, Jin JC, Jiang YW, Du QZ (2014). Improving the Effectiveness of (-)-Epigallocatechin Gallate (EGCG) against rabbit atherosclerosis by EGCG-Loaded nanoparticles prepared from chitosan and polyaspartic acid. J Agric Food Chem.

[143] Ramachandran B, Jayavelu S, Murhekar K, Rajkumar T (2016). Repeated dose studies with pure Epigallocatechin-3-gallate demonstrated dose and route dependant hepatotoxicity with associated dyslipidemia. Toxicol Rep.

[144] Bartikova H, Bousova I, Matouskova P, Szotakova B, Skalova L (2017). Effect of green tea extract-enriched diets on insulin and leptin levels, oxidative stress parameters and antioxidant enzymes activities in obese mice. Polish J Food Nutr Sci.

[145] Chen T, Liu AB, Sun S, Ajami NJ, Ross MC, Wang H (2019). Green tea polyphenols modify the gut microbiome in db/db mice as co-abundance groups correlating with the blood glucose lowering effect. Mol Nutr Food Res.

[146] Al-Shaeli SJ, Watkins AJ, Brown JE (2017). Effect of green tea extract and epigallocatechin gallate on glucose and insulin levels in high glucose fed mice. Diabet Med.

[147] Fu Z, Zhen W, Yuskavage J, Liu DM (2011). Epigallocatechin gallate delays the onset of type 1 diabetes in spontaneous non-obese diabetic mice. Br J Nutr.

[148] Bao S, Cao Y, Fan C, Fan Y, Bai S, Teng W (2014). Epigallocatechin gallate improves insulin signaling by decreasing toll-like receptor 4 (TLR4) activity in adipose tissues of high-fat diet rats. Mol Nutr Food Res.

[149] Catanzaro DP, Mena Laura EE, Cestari TM, Arantes RVN, Garlet GP, Taga R (2018). Green tea prevents vascular disturbs and attenuates periodontal breakdown in long-term hyperglycaemia in T1D rats. J Clin Periodontol.

[150] Chen TS, Liou SY, Kuo CH, Pan LF, Yeh YL, Liou J (2017). Green tea epigallocatechin gallate enhances cardiac function restoration through survival signaling expression in diabetes mellitus rats with autologous adipose tissue-derived stem cells. J Appl Physiol.

[151] Dash RP, Ellendula B, Agarwal M, Nivsarkar M (2015). Increased intestinal P-glycoprotein expression and activity with progression of diabetes and its modulation by epigallocatechin-3-gallate: Evidence from pharmacokinetic studies. Eur J Pharmacol.

[152] Fiorino P, Evangelista FS, Santos F, Magri FMM, Delorenzi J, Ginoza M (2012). The effects of green tea consumption on cardiometabolic alterations induced by experimental diabetes. Exp Diabetes Res.

[153] Li Y, Zhao S, Zhang W, Zhao P, He B, Wu N (2011). Epigallocatechin-3-O-gallate (EGCG) attenuates FFAs-induced peripheral insulin resistance through AMPK pathway and insulin signaling pathway in vivo. Diabetes Res Clin Pract.

[154] Lombo C, Morgado C, Tavares I, Neves D (2016). Effects of prolonged ingestion of epigallocatechin gallate on diabetes type 1-induced vascular modifications in the erectile tissue of rats. Int J Impot Res.

